# Crowdsourcing the Unknown: The Satellite Search for Genghis Khan

**DOI:** 10.1371/journal.pone.0114046

**Published:** 2014-12-30

**Authors:** Albert Yu-Min Lin, Andrew Huynh, Gert Lanckriet, Luke Barrington

**Affiliations:** 1 California Institute For Telecommunications and Information Technology, University of California San Diego, San Diego, California, United States of America; 2 Computer Science and Engineering Dept., University of California San Diego, San Diego, California, United States of America; 3 Electrical and Computer Engineering Dept., University of California San Diego, San Diego, California, United States of America; University of Oxford, United Kingdom

## Abstract

Massively parallel collaboration and emergent knowledge generation is described through a large scale survey for archaeological anomalies within ultra-high resolution earth-sensing satellite imagery. Over 10K online volunteers contributed 30K hours (3.4 years), examined 6,000 km^2^, and generated 2.3 million feature categorizations. Motivated by the search for Genghis Khan's tomb, participants were tasked with finding an archaeological enigma that lacks any historical description of its potential visual appearance. Without a pre-existing reference for validation we turn towards consensus, defined by kernel density estimation, to pool human perception for “out of the ordinary” features across a vast landscape. This consensus served as the training mechanism within a self-evolving feedback loop between a participant and the crowd, essential driving a collective reasoning engine for anomaly detection. The resulting map led a National Geographic expedition to confirm 55 archaeological sites across a vast landscape. A increased ground-truthed accuracy was observed in those participants exposed to the peer feedback loop over those whom worked in isolation, suggesting collective reasoning can emerge within networked groups to outperform the aggregate independent ability of individuals to define the unknown.

## Introduction

Ultra-high resolution satellite imaging enables a new paradigm in global exploration. This study surveys sub-meter resolution satellite imagery of the Mongolian steppe to identify largely undocumented cultural heritage sites across a sparsely populated and undeveloped landscape. With continued advances in sensor technologies, the capabilities and limitations of *remote sensing* is being determined less by data resolution and more by the methods that analyze the increasingly massive datasets. Overwhelming data volumes have often led to automated analytical approaches. However, in visual analytics automated approaches lack the flexibility and sensitivity of human perception when seeking singular, undefined anomalies.

This study therefore utilizes scalable, loosely guided, online volunteer participation to generate human identifications of unknown anomalies within massive volumes of geospatial remote sensing data. The emergence of statistical trends from a large sample of independent inputs highlights the collective human perception of the images' content. Similar to *volunteer geographic information* networks such as OpenStreetMaps [1], inputs from multiple contributors generate a collective map of local knowledge [2], only in this case the term “local” represents the global base of human visual perception.

Turning to the crowd as the “partner of choice” for scalable problem solving is becoming increasingly attractive across broad domains both in science and industry [3], [4]. While many crowdsourcing approaches rely upon gameplay dynamics [5], [6], monetary incentives (e.g., Amazon's Mechanical Turk [7], [8] or [9]) to motivate participation in individual tasks, others utilize the social recognition of charismatic challenges such as space exploration [10] to encourage participation. It has been shown in several of these cases that complex problems, if strategically structured, can be solved by pools of volunteer participants with little to no pre-existing domain knowledge of their analytical challenge [11]. Here we leverage the charismatic challenge of directing a field expedition in search for the tomb of Genghis Khan *(Chinggis Khaan)* to engage tens of thousands of public volunteers and generate millions of micro-contributions towards an archaeological satellite imagery survey. These contributions aggregate into a collective geospatial map of analytical cross-verifications. In this particular case, since there exists no historical or archaeological record describing the physical appearance of this tomb, we turn towards the crowd not only to tackle the data size challenge of large scale satellite remote sensing, but more importantly to pool human perception and intuition when sifting through the data for anything that looks “out of the ordinary”.

This loosely defined search criterium presents a challenge, where a participant's desire for validation or guidance cannot be met with an administrative answer. Thus, we introduce a framework to enable a crowd directed evolution of feature search criteria that mimics crowd reasoning processes in nature. The collective behavior of flocking animals, for example, is enabled through simple decision making and feedback criteria adhered to by the independent agents of that collective [12]. Similar to the vector and spatial referencing data required by individual birds within a flock, our study constructs multi-directional channels of visual reasoning feedback between the individual participant and the crowd. We hope that in this scheme, the collective will guide itself towards an agreement regarding what to look for, essentially facilitating a “collective intelligence” beyond that of individuals whom comprise the collective [13], [14]. We explore this in the case of perceptual reasoning.

## Methodology

### The System

**Figure 1:**
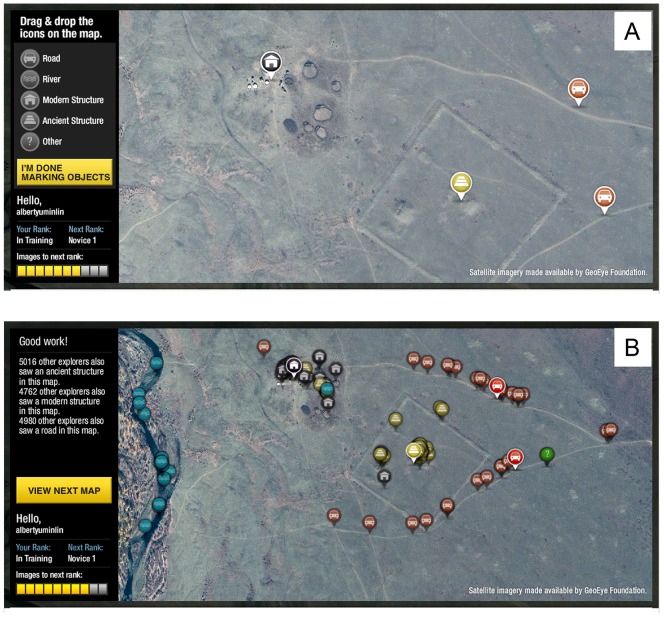
Tagging Interface: (a) Example of tags being made. Tags are color coded with roads (red), rivers (blue), ancient (yellow), modern (grey), and other (green) structures; (b) Example of peer feedback after a participant completes their annotation task. Results of all previous observers of that image tile are shown. Satellite imagery provided courtesy of the GeoEye Foundation.

Ultra-high resolution satellite imagery (0.5 meters/pixel), covering approximately 6,000 km^2^ of landscape was tiled into 84,183 small, semi-overlapping image tiles (1236×630 pixels) and presented to the public via a National Geographic website. There, participants are asked to examine the image tiles in randomized order and identify features from five categories: “*roads*”, “*rivers*”, “*modern structure*”, “*ancient structure*”, and “*other*” (Fig. 1a) by marking up to five “tags” on the image. The random order of the image tasking and the five tag limit per participant for a given image tile were designed to create an equal distribution of influence across all participants, an approach that has been shown to optimize the quality of collective voting [14]. We note that this survey applied multiple satellite images for any given location introducing varying characteristics of time/season-of-collection, color correction, and nadir. This variability may significantly influence on what is visually salient. All images originated from the GeoEye-1 sensor (provided courtesy of the GeoEye Foundation) and underwent pan-sharpening and ortho-rectification, before being presented at native scale.

**Figure 2:**
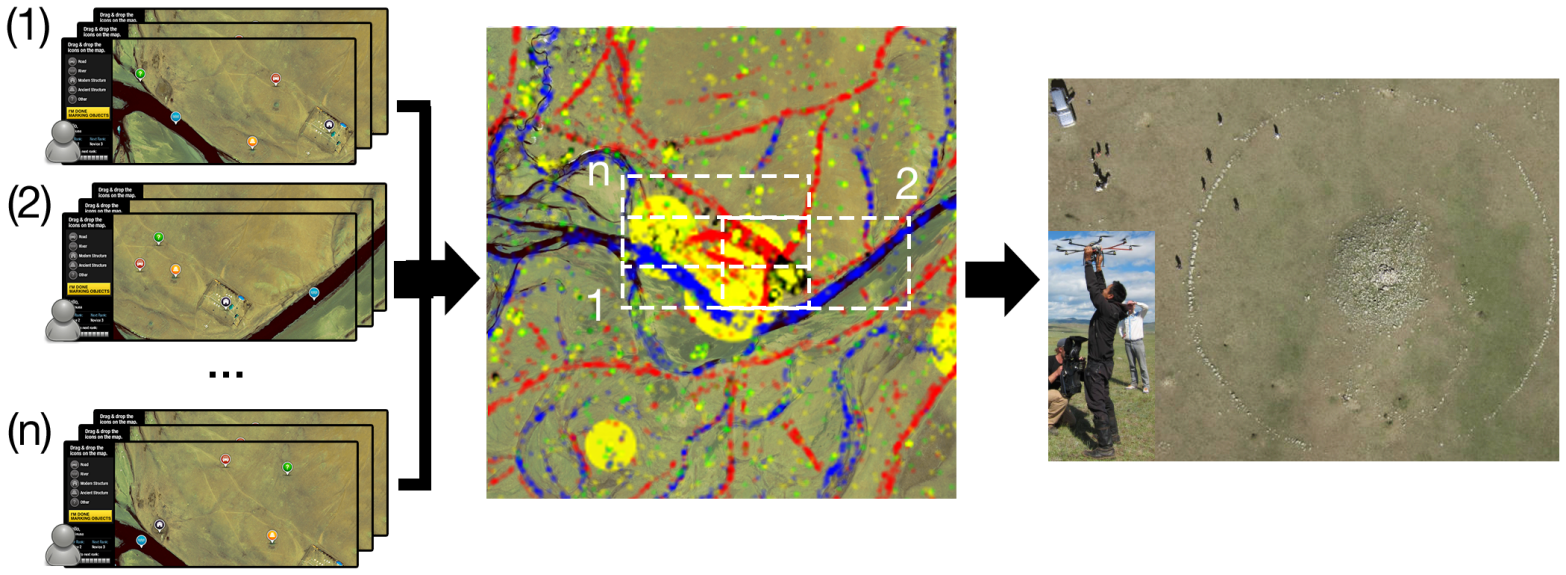
Overall System: *Left* - Participants are provided sub-sectioned imagery tiles in random order to label features such as roads, rivers, and ancient structures through an online interface, we have shown three semi-overlapping tile croppings. *Center* - A geospatial map of observed ground features is generated though the combined input of tens of thousands of independent inputs. Overlapping regions in image tile croppings facilitate a global KDE for a universal comparison of saliency across the entire data set. Regions of highest KDE likelihood (based on user inputs) are highlighted. *Right* - Aerial photography and ground exploration of the location identified by crowd reveals a circular “khirigsuur” burial mound with Bronze Age [24], [25] characteristics. Satellite imagery provided courtesy of the GeoEye Foundation.

After completing a maximum of five tags for a given image tile, and before proceeding to the next randomly selected image tile, participants are shown all tags for that geospatial location previously generated by preceding participants. This provides incremental points of reference to evaluate one's own assessment against that of other peer participants (Fig. 1b). The timing of the anonymous feedback cues was chosen carefully, being presented only after one's tags were committed. This was to avoid the unstable nature of social influence observed in other online markets [15], while still taking advantage of what Krumme et al. [16] recently observed as the informational role cues can play in influencing ones behavior. Furthermore, the participant's completed inputs will contribute as feedback for all future participants. No other form of centralized training is provided, allowing the system to rely upon the emergence of collective consensus or agreement not only to find anomalies but also to generate a constantly evolving search criterion for a target of unknown visual characteristics, the tomb of Genghis Khan. Regions of high agreement among the crowd were ultimately ground-truthed (Fig. 2).

A sub-pool of randomly selected participants were removed from this feedback loop. Upon the completion of a image tile assessment, they would immediately proceed to the next randomly selected image tile. Returning to the flocking analogy, this would be equivalent to “blinders” that would limit sensory data to reference one's movements to neighboring agents. In both cases we depend upon consensus to detect true anomalies on the ground, however from the two pools we can compare collective vs. independent perceptual reasoning in the determination of the unknown search target.

### Extracting Insight from Noisy Data: Kernel Density Estimation

How can we extract usable insight from tens of thousands of independent voices? Human generated data is inherently noisy. Additionally, we must also consider the possibility of random or erroneous contributions. Many factors could motivate or deter sabotage in such open systems [17]. Fortunately, the large volume of parallel independent contribution provides the statistical basis upon which we assess the validity of individual contributions. Essentially, repeated *independent* labeling [18] can provide a mechanism for error correction.

Specifically, we assume that agreement among independent participants is only likely to emerge around observable features and, therefore, that agreement can be used as a metric for validity. In particular, the probability of tag agreement emerging from random choice is statistically low, and the system's constraint of random image tile distribution reduces the possibility of collusion between multiple participants. While this structure facilitates resilience against malicious data manipulation (cooperation), it is noted that the dependency on independent agreement and the even distribution of impact between participants sacrifices the ability to capture value that may come from non-malicious outlier data.

We evaluate crowd consensus as a function of the density of tags. This is characterized through kernel density estimation (KDE) [19], [20], where each tag 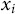 contributes a localized, 2-dimensional Gaussian profile (“kernel”), centered at the location of the tag. The bandwidth of this Gaussian kernel *K* reflects that tag locations may not be precise and ground features extend over multiple pixels. Aggregating these contributions over all tags provided by participants, results in a kernel density function, expressed as:
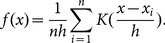
(1)


This function is normalized by *n*, the total number of tags and *h* the kernel bandwidth. *h* is determined by Scotts Rules [21] and corresponds to 20 meters on the ground, aligned with the approximate size of expected archaeological features found in this region (20 meter radius). This kernel density estimate, 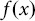, can then be used to assess the likelihood of finding a feature of interest (e.g., a tomb) at every point *x*, in our survey area through 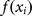. We sampled 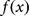 in 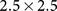 meter cells or 5×5 pixels for computational efficiency.

### Calibrating for Local Effects of Partitioning to Create a Global Data Set

The UI/UX through which participation occurs will significantly impact what insight is collectively extracted from the imagery. To partition and structure our problem, tags were collected through observations on sub-sectioned image tiles, presented in random order and covering only a fraction of the overall survey area. While segmenting the imagery is necessary for the parallel participation framework we devise, we must ultimately account for the local effects of sectioning when combining the tags contributed for each image tile.

Specifically, the random order of image tile distribution to participants results in varying numbers of views from one tile to the next, affecting the density of potential tags and the subsequent probability of emergent agreement. Also, as image tiles are segmented into semi-overlapping rectangles, an object/feature may be presented with variant saliency in more than one image tile based on its position and/or context (with other features) between croppings (Fig. 2). Interestingly, we can take advantage of these overlapping regions to calibrate multiple tiles around shared features.

We begin by determining 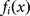, the KDE for each individual image tile *i* using Equation 1. We then minimize the variance between these tile-specific estimates in overlapping regions (50% overlap was produced by the initial tiling of the large satellite image) using least-squares optimization (Equation 2) across all overlapping pairs of tiles: 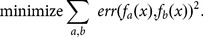
(2)


By defining a function 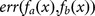 (Equation 3) to represent the discrepancy in density estimates between a pair of overlapping image tiles 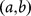, the least-squares approach will attempt to find the set of weights 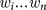 that minimizes the variance in each respective overlapping region:
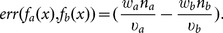
(3)


We determine discrepancy between overlapping tiles pairs through measurable parameters, including 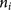, the number of tags collected for tile *i*, and 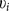, the number of participants who viewed tile *i*. The weighting 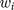 for tile *i*, is an introduced variable for calibration; in particular, they scale the KDE values of each respective tile to minimize the computed variance (Equation 3) between overlapping regions. The weights are determined by least-squares such that Equation 2 is minimized across all overlapping pairs of tiles (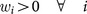). Applying this across the entire data set provides a *global* KDE 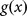 (Equation 4) that ranks individual features based on a universal comparison of saliency. This ranking, discussed bellow, is critical to determine the highest convergence points across the entire crowdsourced dataset, the necessary final step in converting millions of individual inputs into a collective insight.
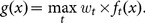
(4)


### Seeing Consensus: Creating Actionable Information

**Figure 3:**
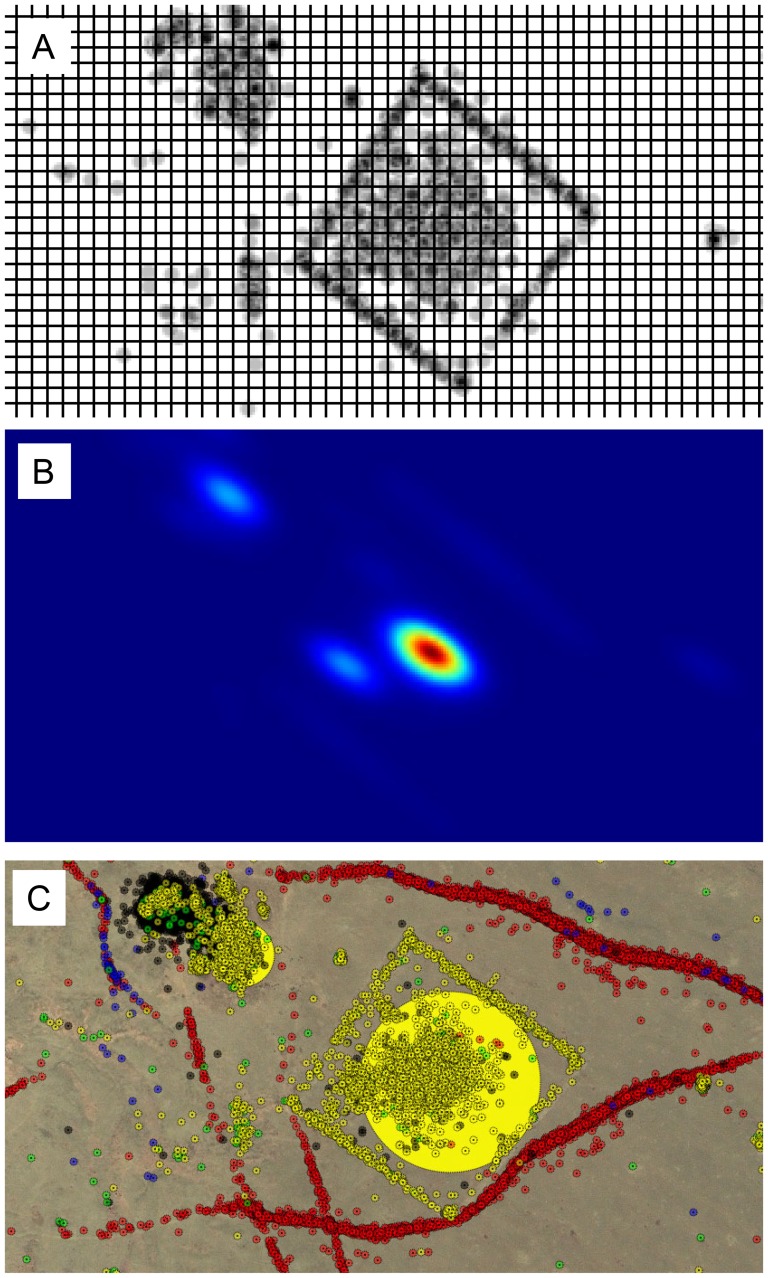
Tag Clustering: (a) Crowd generated tags (grey) within a 2 dimensional space (white) on a 5×5 grid; (b) Kernel density visualized through a color gradient where high density is represented as red and low density as blue; (c)Tag clusters are annotated with semitransparent circles of variable radii, determined as a dependent function of the normalized kernel density estimate of a cluster's center. Large radius circles represent a location of high agreement across the all participants. Satellite imagery provided courtesy of the GeoEye Foundation.

We identify possible ground features from “agreement regions” of high likelihood in the KDE space (described above). Fig. 3 shows (a) crowd generated tags upon a rectangular anomaly and (b) the subsequent kernel density estimation as calculated from Equation 1 represented through a color gradient in which red represents the highest value and blue the lowest value.

When comparing regions of high density across our dataset we wanted to preserve contextual clues in the underlying satellite images while also representing those regions in a highly salient way. We annotated global KDE maxima with semitransparent circles with dimensions depending on the global density estimate as follows:
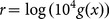
(5)


The function was chosen to slowly increase the radius, *r*, of the circle as the global KDE value, 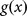, at some coordinate increased. This is shown in Fig. 3 for the same sample archaeological anomaly presented in Fig. 1, where the radius of the circle is scaled with the kernel density estimate at that location with a maximum threshold so that the circles do not overwhelm other locations.

### Ground-truth Validation

The global KDE map provided our field expedition to Northern Mongolia with a ranked list of anomaly identifications to be surveyed on foot and horseback for archaeological validation. Ranking is determined by the global kernel density estimate, from highest to lowest density. Higher ranking locations were ground-truthed, with some exceptions due to extreme physical inaccessibility. This survey combined multi-spectral unmanned aerial remote sensing along with magnetic, electromagnetic, and radar based geophysical scans [22]. This data was subsequently combined within a 3D virtual reality environment [23].

## Results

**Figure 4:**
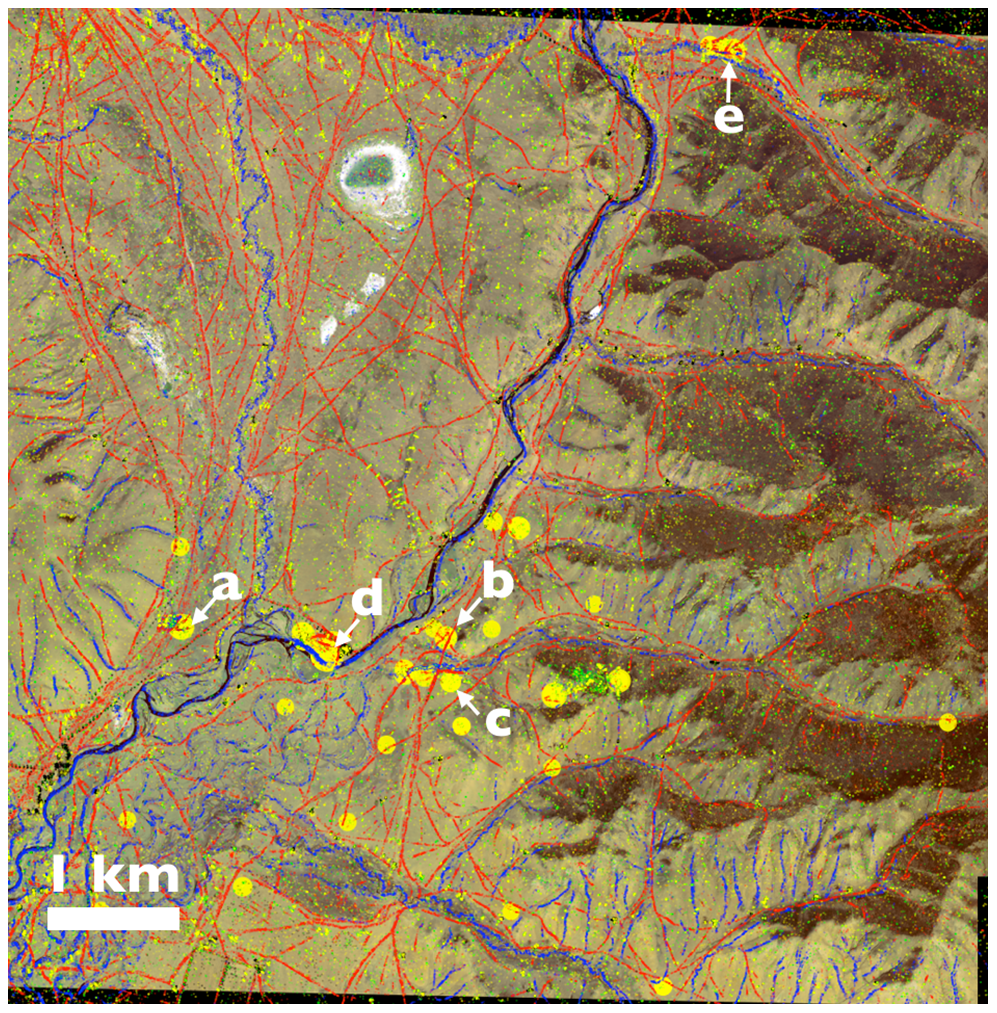
Data Collection Results: Hundreds of thousands of tags overlaid on a subsection of the search area to generate maps of roads (red) and rivers (blue), and to locate ancient (yellow), modern (grey) and other (green) structures. Locations of high agreement (global KDE) in the “ancient structures” category are represented with increased radius. Five example positive identifications are highlighted with labels Fig. 5a- 5e. To construct this figure, we computed the global KDE and applied our visualization function on density peaks across the region. Satellite imagery provided courtesy of the GeoEye Foundation.

The “virtual exploration” system was launched on June 10, 2010 and after 90 days, 5,838 participants had contributed over 1.2 million inputs. After 6 months over 10K participants had generated over 2.3 million inputs (tags), creating a geospatial map that highlighted regions of crowd consensus among the inherently noisy data. Driven by the charismatic opportunity to search for the tomb of Genghis Khan, volunteer participants donated a combined total of 30,000 hours (3.4 years) of human visual analytics (calculated from user interaction time logs). Fig. 4 provides a subsample of the overall crowd generated data (approximately 20% of the total survey area).

Networks of roads (represented in red), and rivers (represented in blue) become clearly outlined by the linear progression of hundreds of thousands of individual and independent tags (Fig. 4). The collective tags provide a remarkably complete map, outlining features with high sensitivity. The region is largely undeveloped and lacks paved roads, thus the red tracks represent the delineation of small, nonuniform and non continuous dirt pathways. Similar detection sensitivity is observed for river delineations.

Regions of high density for both the ancient structure (represented in yellow) and modern structure (represented in grey) categories emerge around localized point features. Interestingly, there exists a high variability in the percentage of tag agreement between categories. Based upon high density regions discovered using kernel density estimation, we observe that 83% of the modern structure tags are part of a high density region (defined as having a non-negligible global KDE value of 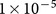, the density estimate of two tags in the same pixel) in comparison to 36.2% of ancient structure tags, 73.6% of river tags, 49.9% of road tags, and 39.9% of “other” tags. As high density regions are a representation of crowd consensus, the results indicate that out of the five available tag categories, participants were least likely to converge upon an agreement when it came to the identification of ancient structures. This was to be expected as we deliberately provide little archaeological context for the definition of an ancient structure, due to the lack of reference knowledge surrounding the tomb's visual appearance. However, where independent agreement did emerge we can observe the collective perception of the crowd, that an “out of the ordinary” feature with potentially ancient origins exists. As stated earlier, we employ the crowd not only to tackle the overwhelming scale of analytics required from our large data set, but to utilize a global human perceptive knowledge base in defining the unexpected.

**Figure 5:**
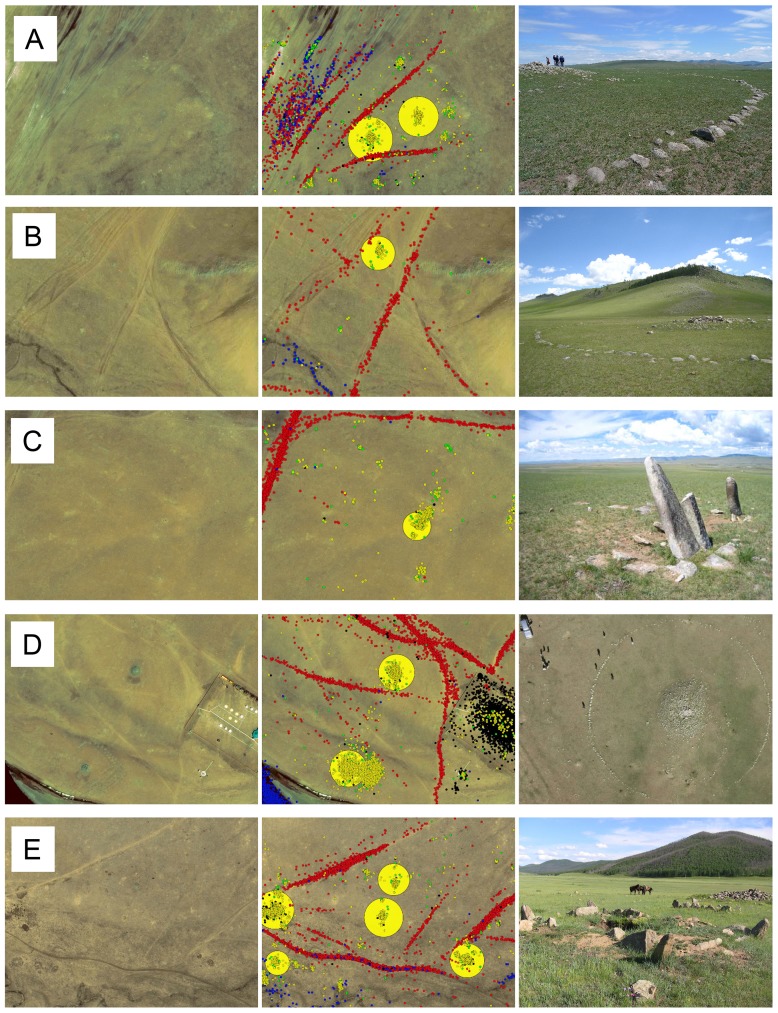
Example Positive Identification (reference Fig. 4): (a) circular “khirigsuur” burial mounds gKDE value: 1.82×10^4^); (b) circular “khirigsuur” burial mounds (gKDE value: 0.29×10^4^); (c) “deer stones” (gKDE value: 0.25×10^4^); (d) rectangular burial mounds (gKDE value: 0.73×10^4^); (e) rectangular burial mound (gKDE value: 0.58×10^4^). Shown archaeological features have early to late Bronze Age origins [24], [25]. Global KDE visualizations for each location were scaled down to fit within the tile. Satellite imagery provided courtesy of the GeoEye Foundation.

Guided by the crowdsourced map, the field team explored and documented one hundred of the highly ranked locations across this extensive landscape. Highly ranked locations that were physically inaccessible due to the rugged nature of the terrain were left un-surveyed. Of the surveyed locations, fifty five archaeologically and culturally significant sites were positively identified including Bronze Age “khirigsuur” burial mounds, “deer stone” megaliths, ancient city fortifications, Tengriist Ovoos and Mongol period burials [24]–[26]. In total, a wide variety of archaeological material was identified from the crowd based analytics, ranging from 10 meter diameter rock piles to 200 meter wall structures. While the specific archaeological nature of these identifications will be discussed in detail elsewhere, five examples of positive identifications are labeled a-e in Fig. 4 and shown in detail in Fig. 5.

**Figure 6:**
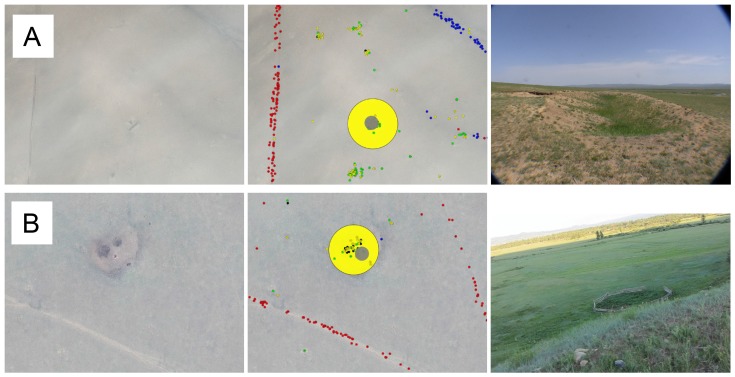
Example False Positives Identification: (a) military tank training trench; (b) nomadic herder's coral. Satellite imagery provided courtesy of the GeoEye Foundation.

False positive identifications, (Fig. 6 a-b), in the tag category of ancient structure occasionally occurred, often due to categorical misinterpretations of other features, i.e remains of modern nomadic settlements or geological formations. While these errors did occur, they were centered around existing observable features and did not represent erroneous random cluster generation. Here we believe the repeated independent and parallel labeling schema for any given image tile, and the random order of image tile distribution over tens of thousands of participants played an important factor in restraining coordination/sabotage.

### Collective vs. Independent Reasoning

This effort seeks “a needle in a haystack”, but in a scenario where the appearance of the needle is undefined. Specifically, not a single burial of the Mongolian imperial family has been identified, and thus we cannot predefine a visual search criterium. We have turned towards the crowd to not only tackle the data size challenge associated with ultra-high resolution satellite imagery, but more importantly to leverage the power of human perception in a search for the unexpected. We have constructed a system of incremental collaborative peer feedback to facilitate a self-evolving collective understanding of what to look for, as explained in previous sections. However, a control group of randomly selected individuals did not participate in the feedback loop, working on the same imagery but in complete isolation. The results from both populations depend upon the emergence of multi-user agreement to locate features, however the two populations experience a fundamental difference in how participants evolve a perceptual understanding of the search target's potential appearance. Here we observe the variance of accuracy between *collective* (feedback) and *independent* (no feedback) populations within data containing ancient sites that have since been validated (ground-truthed). We suggest this can provide some insight into collective reasoning as it is experienced in distributed systems.

### Participant Accuracy

Assuming that each participant's accuracy is independent and normally distributed for both the collective and independent populations, we can compute a mean and standard deviation for both populations and observe any statistically significant differences between them.

**Figure 7:**
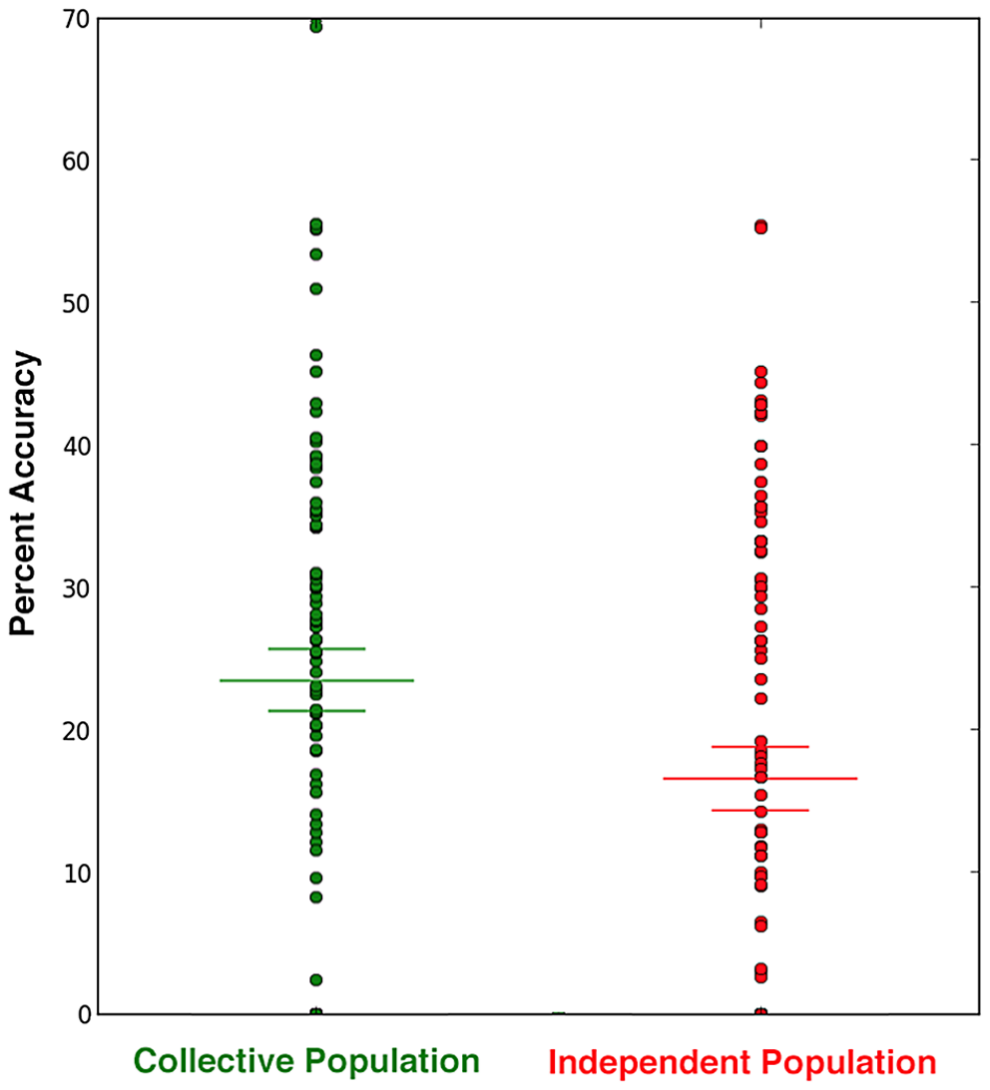
Collective vs. Independent Accuracy: Results from crowdsourced surveys of 100 image tiles containing one or more confirmed archaeological sites are shown as a scatterplot of data points from 0 to 70 (the observed bounds of accuracy) where accuracy is plotted along an individual axis for collective (green) and independent (red) populations. To highlight the discrepancy between the two populations, we have indicated the median accuracy for both the collective (23.6%) and the independent (16.7%) populations.

A group of analysts that had physically ground-truthed locations in Mongolia annotated each image tile that pertained to confirmed archaeological sites by drawing discrete bounding polygons around anything “ancient”. This “true” data set provides the benchmark from which we can determine participant accuracy. Tags within the bounds of an ancient site are considered “accurate tags”. The accuracy for a given tile can be calculated as the number of observed accurate tags normalized by the total number tags for a given population. We calculate this separately for the tags generated from the two populations (collective & independent) for 100 tiles that contained one or more confirmed archaeological sites, the results of which are plotted in Fig. 7.

Applying a simple statistical significance test, we observe a distinct separation between the two data sets. The median percent accuracy of the collective population vs. independent population is 23.6% compared to 16.6%, respectively. This is an observed 42% increase in overall accuracy from those who were subjected to the incremental peer feedback loop (collective reasoning) over those who worked in isolation (independent reasoning). Furthermore, of the 100 tiles, 70 tiles show higher accuracies for collective populations over independent populations.

This suggests that observation based social learning occurs, where a feedback from one's peers could impart knowledge that would translate into the next set of decisions one makes, within a cycle of decisions and feedback, ultimately effecting the ability of the group. However, Rahwan et al. has recently described a *unreflected copying bias* which considers analytical reasoning and one's tendency to copy a positive peer outcome rather than the analytical process required to arrive at that outcome [27]. Even if this is the case, we are depending on the peer feedback loop to facilitate the emergence of the perceived search criteria, articulated through points of independent convergence. The ability to predictably align one's next input with this convergence is, in our case, what defines a positive outcome for that individual. Thus, here “collective reasoning” manifests through the participant's tracking/contribution of a constantly evolving, peer generated, search criteria defined by points of convergence.

The variability in the two subgroups (collective vs. independent) indicates that this is in fact occurring. Furthermore, the reasoning which emerges is statistically more accurate than the results of individuals who were not shown any form of feedback. In essence, the pool of global knowledge can be curated at an individual level through peer based filters that allow us to reason more effectively as a collective.

## Discussion

We have described a crowd based solution to a satellite imagery remote sensing challenge of both data volume and search target ambiguity. Specifically, we charged an online crowd of volunteer participants with the challenge of finding the tomb of Genghis Khan, an archaeological enigma of unknown characteristics widely believed to be hidden somewhere within the range of our satellite imagery. This is a needle in a haystack problem where the appearance of the needle is unknown. To address this constraint we designed a system where participants actively evolve the collective training base of user feedback with their own inputs. Furthermore, the framework of the system created a resilient and self-validating data source through massively paralleled and constrained user inputs. Thus, we rely upon the emergence of agreement regions from independent tags to guide both the online volunteer community as well as the field archaeological expedition that surveyed anomalies on the ground. The entire data set is distributed in randomly chosen image tile subsets to participants, thus a “global” kernel density estimation approach is introduced to normalize saliency across all image tiles and to create an overall agreement region ranking. This concept could be applied as a distributed voting framework, where overlapping subsets allow for large data sets to be subdivided and parsed among many voters and then recombined into a single collective vote.

Of the top 100 accessible locations identified by the crowd, 55 potential archaeological anomalies were verified by the field team, ranging from bronze age to Mongol period in origin.

Yet, the question remains: could these results have been obtained just as effectively or more effectively without crowdsourcing? Or more specifically, could a small team of trained archaeologist have found the anomalies quickly by visually scanning the images on their own? After all we did expend 30K hours (3.4 years) of collective human survey effort.

Looking first at the data size challenge, we have surveyed a historically significant area of roughly 6,000 km^2^. This is twice the size of Yosemite National Park, with equally diverse geologies and significantly greater in-accessibility. A ground survey of this detail for the entire range would have been prohibitive. Yet, at 0.5 meter/pixel resolution, a satellite imagery survey of the same area is in itself exhausting. A single archaeologist would have had to scroll through nearly 20,000 screens (assuming 1280×1024 screen resolution) before covering the whole area.

But putting the data size challenge aside for a moment, we can observe the crowd's ability to be sensitive yet flexible. We continue to emphasize that very little is known about the likely visual appearance of the search target. Thus, we cannot limit our search criteria to what is traditionally “expected” from the known literature. Here is where the authors believe the power of crowdsourcing lies not only in harnessing parallel networks for scalable analytics, but in forming the collaborative frameworks necessary to cultivate collective reasoning. We depend on the crowd to process and identify the unexpected.

Within our framework we observed that when participants were not provided the incremental peer based feedback loop they were statistically less likely to positively identify these anomalies, suggesting a form of collective reasoning has emerged within our participant pool that is variant and potentially more effective than the accumulated independent reasoning of individuals within that pool. Furthermore, while we acknowledge that there may be anomalies that remain undetected, this statistical variance suggests that largely parallel analytics (crowdsourcing) can provide better outcomes than an individual survey.

While this study focused on an archaeological survey, there exists a broad range of challenges where scalable human perception networks could be effectively applied. These concepts have been further explored in applications ranging from humanitarian response to search & rescue [28]–[30]. The activities have not only tapped into our connectivity to scale human analytics, but also for the social mobilization of human attention [31], [32]. A recent direct derivative of the effort described here can be seen in Digital Globe Inc.'s “Tomnod” (Mongolian word meaning “big eye”) survey for the missing Malaysia Airlines flight MH370, where over 8 million participants surveyed over 1 million km^2^ of ultra-high resolution satellite imagery for anomalies. The shear mass of participation in this example provides a glimpse of the potential of our networked society.

These crowdsourcing activities help us dive into the unknown and extract the unexpected. However, beyond that they present a fundamentally new construct for how we, as a digitally connected society, interact with information. The ability to focus and route networks of human attention at such massive scales, coupled with the functional ability for meaningful micro-contributions at individual scales, presents yet another evolutionary step in our collective ability to reason.
