# Correction: Crowdsourcing the Unknown: The Satellite Search for Genghis Khan

**DOI:** 10.1371/journal.pone.0121045

**Published:** 2015-03-25

**Authors:** The PLOS ONE Staff

The first author is cited incorrectly. The author should be cited as Lin AYM. The full citation should be: Lin AYM, Huynh A, Lanckriet G, Barrington L (2014) Crowdsourcing the Unknown: The Satellite Search for Genghis Khan. PLoS ONE 9(12): e114046. doi:10.1371/journal.pone.0114046
